# NMR and Patch-Clamp Characterization of Yeast Mitochondrial Pyruvate Carrier Complexes

**DOI:** 10.3390/biom13050719

**Published:** 2023-04-22

**Authors:** Zhen Wang, Wen Ding, Maosen Ruan, Yong Liu, Jing Yang, Huiqin Zhang, Bing Shen, Junfeng Wang, Yunyan Li

**Affiliations:** 1 High Magnetic Field Laboratory, CAS Key Laboratory of High Magnetic Field and Ion Beam Physical Biology, Hefei Institutes of Physical Science, Chinese Academy of Sciences, Hefei 230031, China; minioreo@mail.ustc.edu.cn (Z.W.); maosen@hmfl.ac.cn (M.R.); yongliu6@mail.ustc.edu.cn (Y.L.); yj91@hmfl.ac.cn (J.Y.);; 2 Hefei Institutes of Physical Science (Branch of Graduate School), University of Science and Technology of China, Hefei 230026, China; 3 School of Basic Medical Sciences, Anhui Medical University, Hefei 230032, Chinashenbing@ahmu.edu.cn (B.S.); 4 Institutes of Physical Science and Information Technology, Anhui University, Hefei 230601, China

**Keywords:** Mpc, PRE, NMR, patch-clamp, oligomeric state, protein interactions

## Abstract

The mitochondrial pyruvate carrier (Mpc) plays an indispensable role in the transport of pyruvates across the mitochondrial inner membrane. Despite the two distinct homologous proteins, Mpc1 and Mpc2, were identified in 2012, there are still controversies on the basic functional units and oligomeric state of Mpc complexes. In this study, yeast Mpc1 and Mpc2 proteins were expressed in a prokaryotic heterologous system. Both homo- and hetero-dimers were successfully reconstituted in mixed detergents. Interactions among Mpc monomers were recorded utilizing paramagnetic relaxation enhancement (PRE) nuclear magnetic resonance (NMR) methods. By single-channel patch-clamp assays, we discovered that both the Mpc1–Mpc2 hetero-dimer and Mpc1 homo-dimer are able to transport K^+^ ions. Furthermore, the Mpc1–Mpc2 hetero-dimer demonstrated the ability to transport pyruvates, at a rate significantly higher than that of the Mpc1 homo-dimer, indicating that it could be the basic functional unit of Mpc complexes. Our findings provide valuable insights for further structural determination and the study of the transport mechanism of Mpc complexes.

## 1. Introduction

Pyruvate occupies a pivotal metabolic branch point in the regulation of carbon homeostasis [1]. It is the final product in the glycolysis pathway [1,2] which can be converted to a lactate in cytosol [3], or alternatively transported into the mitochondrial matrix, where it is either converted into an oxaloacetate by pyruvate carboxylase (PC) to facilitate gluconeogenesis, or oxidized into acetyl-CoA by the pyruvate dehydrogenase complex to initiate the TCA cycle [3,4]. Because pyruvate is a key metabolic intermediate in those biological metabolism pathways, multitudinous metabolic disorders including neurogenerative disease, diabetes, heart disease and cancer are associated with aberrant pyruvate metabolism [3,5].

The mitochondrial pyruvate carrier (Mpc), located in the inner mitochondrial membrane, is critical for transporting pyruvate from cytosol into the mitochondrial matrix. Although the existence of Mpc was predicted biochemically in the 1970s [6,7], the process of identifying its molecular components has taken several decades to unravel. In 2012, a crucial breakthrough was made in this field in that two distinct homologous proteins, Mpc1 and Mpc2, were identified to form a complex that is essential for mitochondrial pyruvate transport [6,7]. Mpc1 and Mpc2 are evolutionarily conserved between yeasts and human-beings [3], at molecular weights from 12 kDa to 15 kDa. In yeasts, the Mpc family includes another Mpc isoform named Mpc3, which has about 79% of the same amino acids as Mpc2 has [1].

Though significant advancements have been made since the initial discovery of the Mpc complexes in the 1970s [6,7], there are still some challenges, such as the verification of basic functional units and oligomer states, and the determination of their 3D structure. In mammalian cells, Mpc1 and Mpc2 are both required for pyruvate transport, and the Mpc complex is seemingly composed merely of heterotypic Mpc1 and Mpc2. In contrast, in yeasts, Mpc1 is the core subunit, Mpc2 and Mpc3 are regulatory units, and the switch between the Mpc1–Mpc2 oligomer and Mpc1–Mpc3 oligomer mainly depends on the carbon source in the media of yeasts [8,9]. Additionally, yeasts grow slowly without Mpc1 and without amino acids in the medium [10], and meanwhile, the capacity of pyruvate transport declines [1]. Available evidence suggests that Mpc homo-oligomers have individual activities. As reported, Mpc1 exhibits diverse expression levels in different cells. Mpc2 has been clarified experimentally as an autonomous pyruvate transporter [11]. Additionally, it was demonstrated that Mpc2 was closely associated with hepatic gluconeogenesis in the liver [12,13,14]. According to Lee et al., it is highly likely that the dominant type for pyruvate transport is the hetero-dimer even though the hetero-dimer of human Mpc can co-exist with homo-dimers [11,15]. Currently, the heterotypic Mpc complex is considered the prevailing viewpoint [16].

To address the problem of functional constitution and the oligomeric state of MPC proteins, herein, we utilized the relatively simple prokaryotic expression system. Moreover, homo- and hetero-dimers were successfully assembled into mixed detergent micelles by the denaturation–refolding method, and their oligomeric state and the protomer stoichiometry were characterized by paramagnetic resonance enhancement (PRE) nuclear magnetic resonance (NMR) and biochemical methods. Our study shows that both homo- and hetero-dimers can form in vitro, with the hetero-dimer exhibiting greater stability. Furthermore, the patch-clamp technique was also used to record the transport capacity of homo- and hetero- MPC dimers to transport pyruvate as well as small ions such as K^+^. We find that the hetero-dimer is capable of transporting pyruvate, whereas the homo-dimer does not have this ability, despite being able to transport K^+^ ions.

## 2. Materials and Methods

### 2.1. Plasmid Constructions

Genes encoding Saccharomyces cerevisiae Mpc1 (NCBI reference sequence: NM_001180945) and Mpc2 (NCBI reference sequence: NM_001179293) were cloned into an optimized pET28a vector with a C-terminal 8×His-tag by polymerase chain reaction (PCR) using the Saccharomyces cerevisiae cDNA library as a template. For Mpc1, the forward primer and reverse primer are 5′-GGAATTCCATATGTCTCAACCGGTTCAACGCGCTG-3′ and 5′-CGCGGATCCCTGTTTACCAGTTTTTTCTTTCTCTTTCCA-3′; for Mpc2, the forward primer and reverse primer are 5′-GGAATTCCATATGTCTACATCATCCGTACGTTTTGCATTT-3′ and 5′-CGCGGATCCTCTGCCCGTAGTAATTTCCTTTTT-3′. Genes that were tagged with Mpc1′s C-terminal, composed of ENLYFQG (the TEV enzyme recognition site), flexible linker (GGGGS)_4_WSHPQFEK and 8×His-tag, were shortly named Mpc1^linker^ and were synthesized by Tsingke Biotechnology Co., Ltd. through the Gibson Assembly [17].

### 2.2. Protein Expression and Purifications

*Escherichia coli* (*E. coli*) Rosetta 2 (DE3) cells (Novagen) were transformed with all the Mpc protein constructs. Cells were cultured in the Luria-Bertani (LB) medium at 37 °C until the optical density at 600 nm (OD_600_) reached 0.8, and then were induced with 0.9 mM isopropyl β-D-1-thiogalatopyranoside (IPTG) at 31 °C for 14 h. The isotopically labeled proteins were expressed in cells cultured in an M9 medium containing ^15^NH_4_Cl (0.5 g/L) as the sole nitrogen source for NMR analysis. From Cambridge Isotope Laboratories, Inc., in Tewksbury, Massachusetts, United States, ^15^N-NH_4_Cl was purchased.

After cell lysis, through a high-pressure homogenizer (Union-Biotech (Shanghai) Co., Ltd. Shanghai, China) at 800 psi, all targeted proteins in their inclusion body form were dissolved in a chaotropic buffer (50 mM Tris/HCl (pH 8.0), 0.5 M NaCl, 5 mM β-ME, 10 mM imidazole and 6 M guanidine hydrochloride (Gdn-HCl)) with rotators rotating overnight. After the suspension turned translucent, the inclusion body was purified via nickel-nitrilotriacetic acid affinity chromatography (Ni-NTA chromatography) in a washing buffer (50 mM Tris/HCl (pH 8.0), 0.5 M NaCl, 6 M Gdn-HCl and 5 mM β-ME) of imidazole at increasing concentrations (10 mM, 30 mM and 1 M). The washing mixture of 1 M imidazole was then collected for stepwise dialysis in 10 kDa pore-size dialysis membranes (Beijing Labgic Technology Co., Ltd. Beijing, China). The dissolved inclusion body required about 14 h to entirely aggregate and to form a relatively pure precipitate. All of the precipitate was dissolved in 8 M Gdn-HCl before being refolded in the refolding buffer (50 mM Tris/HCl (pH 7.0), 300 mM NaCl, 500 mM arginine, 0.5 mM oxidized glutathione, 5 mM reduced glutathione, 1 mM EDTA, 10 mM β-ME and 10% glycerol with 5 mM N, N-dimethyldodecylamine N-oxide (LDAO, Anatrace Products LLC, Maumee, Ohio, United States) and 2.5 mM n-Dodecyl phosphocholine (DPC, Anatrace Products LLC, Maumee, Ohio, United States) as the surfactants. Afterwards, proteins were further purified utilizing a Superdex 75 column (GE Healthcare, Piscataway, NJ, USA) in a size exclusion chromatography (SEC) buffer of 100 mM Tris/HCl (pH 7.5), 150 mM NaCl, 1 mM EDTA, 10 mM β-ME, 1 mM DPC and 2 mM LDAO. The purity of the protein was analyzed by SDS-PAGE. Protein concentrations were estimated using the molar absorption coefficient and via absorbance spectroscopy.

### 2.3. Size-Exclusion Chromatography with Multi-Angle Light Scattering (SEC-MALS)

SEC-MALS analysis was performed with a Superdex 75 column (GE Healthcare, Piscataway, NJ, USA) coupled in line with a light scattering detector (Dawn HELEOS II, Wyatt Technologies). The SEC-MALS/UV/RI measurement was used to determine each Mpc complex molecular mass based on the three-detector method [18,19]. Every single Mpc complex was injected at 0.3 mL/min into the Superdex 75 column equilibrated with 100 mM Tris/HCl (pH 7.5), 150 mM NaCl, 1 mM EDTA, 10 mM β-ME, 1 mM DPC and 2 mM LDAO. All data were recorded and analyzed with ASTRA 7.0.1 (Wyatt Technologies).

### 2.4. NMR Experiments

NMR samples were prepared as above with a SEC buffer containing 25 mM NaAc (pH 6.0), 50 mM Glutamic acid, 50 mM Arginine, 10 mM β-ME, 1 mM EDTA, 1 mM DPC, and 2 mM LDAO. For inter-chain PRE measurements, cysteines of the Mpc proteins were labeled with the commonly used paramagnetic probe S-(1-oxyl-2,2,5,5-tetramethyl-2,5-dihydro-1H-pyrrol-3-yl) methyl methanesulfonylthioate (MTSL) [20]. After the MTSL-labeled monomer renaturated with a ^15^N-labeled monomer, the PRE effects were observable in 2D ^1^H-^15^N band-selective excitation short-transient transverse relaxation-optimized spectroscopy (BEST-TROSY). In order to acquire monomers that were labeled with MTSL, the subsequent steps were implemented: the unlabeled Mpc proteins were dissolved in 6 M Gdn-HCl and subsequently treated with a final concentration of 20 mM 1,4-dithiothreitol. After 4 h of incubation, the 1,4-dithiothreitol was eliminated by dialysis. MTSL was added to the solution at a ratio of 20 to 1 when it was shielded from light. The solution was then chilled overnight at 4 °C. To eliminate extra MTSL, additional dialysis was conducted. To obtain the spectrum of a diamagnetic sample, Vitamin C (VC) was added overnight during incubation to halt the reaction between MTSL and cysteines. All NMR spectra were conducted at 308 K using a Bruker 850 MHz NMR spectrometer equipped with a 5 mm CryoProbe. Bruker TopSpin 3.6.2 was used to display and analyze all the data.

### 2.5. Circular Dichroism Spectroscopy

The secondary structure and thermostability of protein complexes were investigated using circular dichroism (CD) spectroscopy on J-1700 Circular Dichroism Spectrophotometer. The protein complexes were prepared in a phosphate buffer (consisting of pH 8.0, 3.52 mM KH_2_PO_4_, 46.48 mM K_2_HPO_4_, 50 mM K_2_SO_4_, 2 mM LDAO and 1 mM DPC) for analysis at room temperature using Spectra Analysis software (JASCO) and for variable temperature CD analysis using the temperature interval measurement function on the JASCO instrument.

The spline interpolation method was used to compute the areas between the two temperature–CD curves (25 °C and 95 °C) to quantify the thermostability. In addition, the curve variation magnitudes represented by delta mean residue ellipticity were examined using both the Friedman nonparametric hypothesis test and Kendall’s coefficient of concordance, as both methods are highly suited to non-normally distributed data as nonparametric tests.

### 2.6. Patch-Clamp Assays

All the three Mpc complexes were reconstructed in giant liposomes for a single-channel patch-clamp later. The preparation of giant liposomes was performed mainly based on the previously widely applied protocol [21,22].

The lipid solution was agitated for 20 min with a vortex stirrer (Haimen Kylin-Bell Lab Instruments Co., Ltd.) after 60 mg of POPC was dissolved in 4 mL of a filtered bath buffer (10 mM Mops (pH 7.2) and 250 mM KCl for the K^+^ test or 250 mM pyruvate-N-methyl-glucamine (pyruvate-NMDG) for the pyruvate test). The suspension was sonicated for 10 min under nitrogen protection, then supplemented with 2 mL of 2× bath buffer. Next, 270 μL of a lipid solution was treated with 40 ng of Mpc proteins in 180 μL of the SEC buffer. Dialysis, which was conducted in the bath solution, eliminated the mixed detergents. The precipitate was resuspended in 30 μL of a 15 mM HEPES buffer (pH 7.2–7.4) with 5% (*v*/*v*) ethylene glycol after ultracentrifugation at 160,000× *g* for 1.5 h. After being aliquoted for two coverslips, the resuspension was dried in a box at 4 °C for 10–12 h using silica gel particles as a desiccant. Before the patch-clamp experiment, the dried lipid droplet was rehydrated overnight at 4 °C with 15 μL of the bath buffer. NMDG herein refers to an organic monovalent cation which features a charged methylamine head group and a glucose-like hydrophilic tail, resulting in a linear molecular structure. This structure measures 6.4 Å in width and 12 Å in length, with a mean diameter of approximately 7.3 Å [23]. NMDG blocking in ion channels has been broadly observed [24], which makes it a good substitute for a cation in the patch-clamp assay.

The sample was placed in a tiny flat cylindrical cell with 500 μL of a bath solution. Patch-clamp single-channel recordings of giant liposome membranes were performed at 30–40 MΩ resistance using patch pipettes filled with a pipette buffer (10 mM Mops (pH 7.2) and 250 mM KCl for the K^+^ test or 250 mM pyruvate-NMDG for the pyruvate test) as the solution inside the giant liposomes. The experiment was carried out utilizing the voltage clamp mode on an EPC9 patch-clamp amplifier (HEKA Elektronoik, Lambrecht/Pfalz, Germany), with the bath solution being identical to the solution inside the giant liposomes. The PatchMaster (HEKA) program was used to implement the ramped protocol of depolarization from −150 mV to +150 mV over 1000 ms. The currents were digitized at 0.15 ms intervals and filtered at 0.5 kHz.

## 3. Results

### 3.1. Expression and Purification of Mpc Complexes

**Figure 1:**
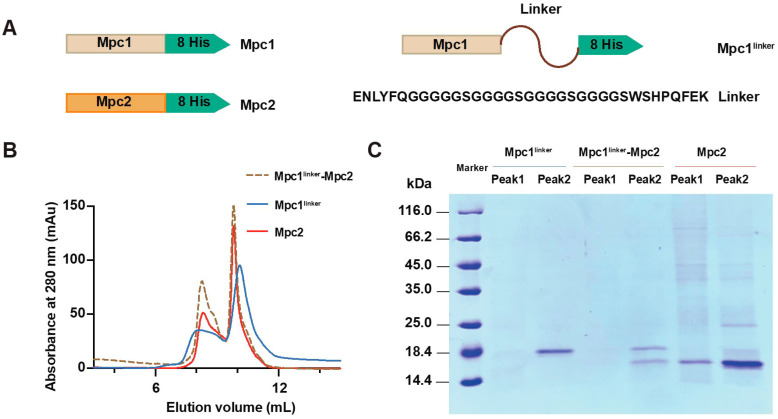
Constructs and biochemical analysis of Mpc proteins. (**A**) Linear overview of the domain organization of the Mpc constructs used in this paper; (**B**) size exclusion chromatography of the Mpc complexes from the Superdex 75 10/300 GL column in the buffer containing 100 mM Tris/HCl (pH 7.5), 150 mM NaCl, 1 mM EDTA, 10 mM β-ME, 1 mM DPC and 2 mM LDAO; (**C**) SDS-PAGE analysis of each elution peak of the three Mpc oligomers in (**B**); the protein bands were detected by Coomassie blue staining.

In this report, we try to express yeast Mpc1 and Mpc2, utilizing prokaryotic heterologous expression, aiming to obtain Mpc1 and Mpc2 homo-complexes, together with Mpc1–Mpc2 hetero-complexes. Because the molecular weights of Mpc1 and Mpc2 are very similar, Mpc1^linker^ was introduced. A linker (GGGGS)_4_ was proven to be flexible enough not to affect the functional and structural modification of the target protein [25]. The constructs of Mpc proteins are shown in Figure 1A.

As mentioned in the Materials and Methods section, the target yeast Mpc proteins were initially purified in a Ni-NTA chromatography column. The following important procedure was to refold the Mpc monomers through drop-wise dilution in a refolding buffer with Mpc1^linker^, Mpc2, and pre-mixed Mpc1^linker^ and Mpc2 at a molar ratio of 1:1, respectively. In order to obtain stable and uniform complexes, L-Arginine [26], glycerol and mixed LDAO/DPC detergents at a ratio of 2:1 were added to the refolding buffer. The refolded Mpc proteins were further purified by size exclusion chromatography utilizing a Superdex 75 Increase 10/300 GL column. The elution volumes of Mpc1^linker^, Mpc1^linker^–Mpc2, and Mpc2 were very similar, all having two peaks (Figure 1B) at around 8 mL and 10 mL. The second peaks were sharp and symmetric, which indicated good protein homogeneity.

SDS-PAGE analysis was conducted on each eluted individual peak, and Figure 1C indicates that the second peaks consisted of highly pure proteins. In particular, Mpc1^linker^–Mpc2 (Figure 1C, lane 5) exhibited two protein bands with a molar ratio of approximately 1:1, as observed via Coomassie brilliant blue staining. Together with the fact that the elution volumes of conalbumin (75 kDa) and ovalbumin (44 kDa) were ~8.8 mL and ~10.0 mL separately (Cytival Manual), we guessed that the second peaks at around 10 mL may have consisted of 2 × n (n = 1, 2) monomers that may have formed complexes. In additionn, no obvious protein bands were found in the first peaks on Mpc1^linker^ and Mpc1^linker^–Mpc2. The above results indicate that the proteins obtained after affinity column purification, inclusion body denaturation, protein refolding and fast protein liquid chromatography were relatively pure; the heterologous recombinant expression of yeast Mpc proteins is feasible.

### 3.2. Characterization of Homo- and Hetero-Mpc Oligomer States

**Figure 2:**
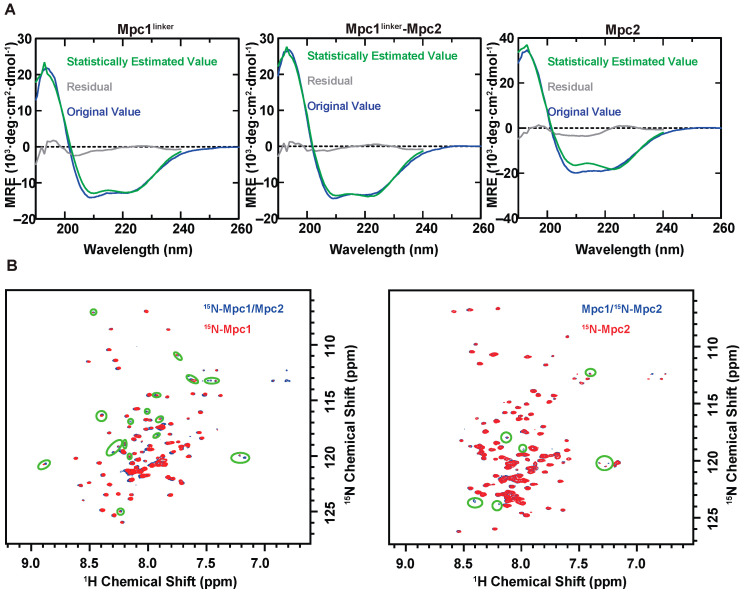
Formation of complexes estimated by CD and NMR experiments. MRE denotes “Mean Residual Ellipticity”. (**A**) CD curves of Mpc1^linker^complex, Mpc2 complex and Mpc1^linker^–Mpc2 complex at room temperature, respectively; the deep blue curves are original values, the green curves are statistical estimated values by temperature interval measurement (JASCO), and the grey curves are the systemic residuals. (**B**) Overlap of the ^1^H-^15^N BEST-TROSY spectra; the differences between ^15^N-Mpc1/Mpc2 and ^15^N-Mpc1, Mpc1/^15^N-Mpc2 and ^15^N-Mpc2 are circled by green elliptical circles.

CD experiments at room temperature were conducted to characterize the above three samples. As shown in (Figure 2A), negative absorption at 208 nm and 222 nm, and relatively pronounced positive absorption at 190 nm, indicated that they were all well folded. To investigate the differences between the secondary structures of the three complexes, the compositions of their secondary structures were estimated (and are shown in Figure S2). All three complexes exhibited an alpha-helix, a turn and a random structure. The alpha-helices in the Mpc1^linker^ and Mpc2 homo-complexes, respectively, accounted for 45.2% and 57.9% of the total composition, which were almost in line with results of the study on monomers of Li et al. [27]. However, one notable difference between Mpc1^linker^ and Mpc2 was the absence of the beta component in the secondary structure of Mpc2, indicating that Mpc1^linker^ and Mpc2 have distinct secondary structures. In Mpc1^linker^–Mpc2, the composition of the alpha-helix accounts for 46.3% of the total composition, which is similar to that in Mpc1^linker^, rather than the simple average of the compositions in Mpc1^linker^ and Mpc2. As shown in Figure S2, the secondary structure is similar to those in Mpc1^linker^, but different from those in Mpc2, suggesting that Mpc1 may play an important role in stabilizing the structure in the Mpc1–Mpc2 complex.

Multifunctional NMR methods were applied to further verify the proteins obtained by the methods. We prepared four samples in a NMR buffer with ^15^N-Mpc1, ^15^N-Mpc2, pre-mixed ^15^N-Mpc1/Mpc2, and Mpc1/^15^N-Mpc2 at a molar ratio of 1:1. High-resolution 2D ^1^H-^15^N BEST-TROSY spectra were successfully obtained as shown in Figure S1. Great chemical shift differences were detected in the BEST-TROSY spectra between ^15^N-Mpc1/Mpc1 and ^15^N-Mpc2/Mpc2, indicating notable differences in secondary/ tertiary structure between the two proteins. These findings are consistent with the results obtained from the CD spectra. Moreover, significant intensity and chemical shift alterations were detected in the BEST-TROSY spectra between ^15^N-Mpc1/Mpc1 and ^15^N-Mpc2/Mpc2, which demonstrated that the state of ^15^N-Mpc1 between ^15^N-Mpc1/Mpc1 and ^15^N-Mpc1/Mpc2 samples is different. A similar phenomenon was recorded for state of ^15^N-Mpc2 in ^15^N-Mpc2/Mpc2 and Mpc1/^15^N-Mpc2 samples. Those results, together with the basic rule that there was a strong correlation between the chemical shifts observed and along with the intensities of each amino acid and its local structure, further indicated the formation of a Mpc1–Mpc2 hetero-complex.

**Figure 3:**
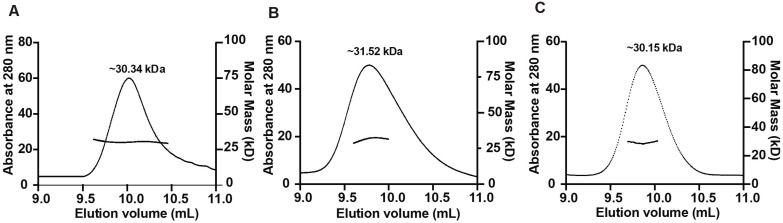
Size exclusion chromatography coupled with multi angle light scattering (SEC-MALS) analysis of the second elution peak of the three Mpc complexes. (**A**) Mpc1 homo-dimer; (**B**) Mpc1–Mpc2 hetero-dimer; (**C**) Mpc2 homo-dimer. The left and right axes represent the UV detector reading and molecular mass, respectively. The horizontal black curves represent the calculated molecular mass, and the average mass of each elution peak of the three Mpc complexes are shown above the UV curves.

To determine the oligomeric states, SEC-MALS experiments were performed. The molecular weights of the three complexes were about 30.34 kDa, 31.52 kDa, and 30.15 kDa for the Mpc1 homo-dimer, Mpc1–Mpc2 hetero-dimer, and Mpc2 homo-dimer, respectively (Figure 3), which matched their dimeric forms, and the molecular weight data points fluctuated very little, revealing the uniformity of the three Mpc complexes.

### 3.3. Analysis of Mpc Monomer Interactions Using PRE NMR

**Figure 4:**
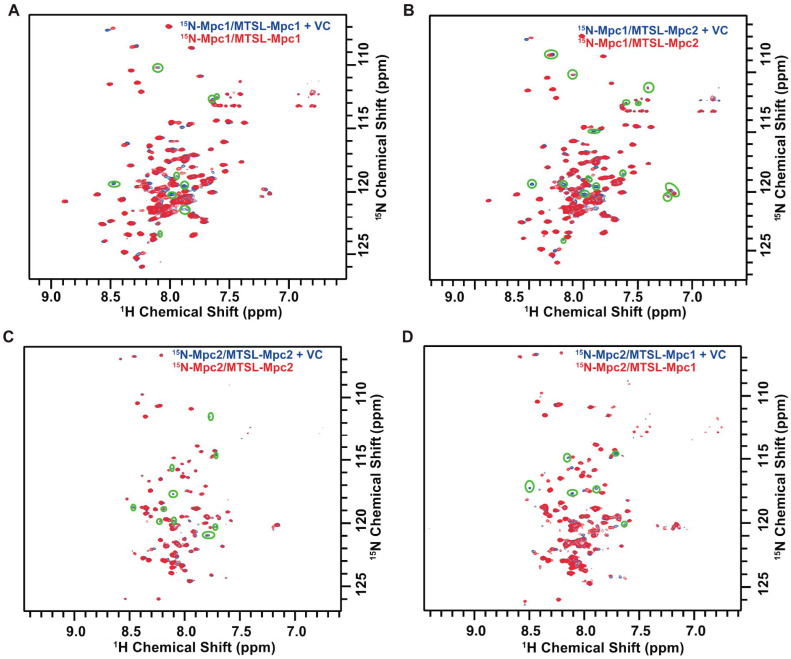
Paramagnetic relaxation enhancement (PRE) experiments. (**A**) Overlap of BEST-TROSY spectra of ^15^N-Mpc1/MTSL–Mpc1 with and without Vitamin C (VC); (**B**) overlap of BEST-TROSY spectra of ^15^N-Mpc1/MTSL–Mpc2 with and without VC; (**C**) overlap of BEST-TROSY spectra of ^15^N-Mpc2/MTSL–Mpc2 with and without VC; (**D**) overlap of BEST-TROSY spectra of ^15^N-Mpc2/MTSL–Mpc1 with and without VC. The broadening peaks are circled by green elliptical circles.

To characterize the interactions between the monomers of a shared dimer, PRE experiments (as shown in Figure 4A–D) were performed. The MTSL was used to label C87 of the Mpc1 monomer (MTSL–Mpc1) and C86 and C111 of the Mpc2 monomer (MTSL–Mpc2), respectively. Subsequently, co-refolding of the MTSL-labeled monomer and the ^15^N-labeled monomer was carried out to obtain the paramagnetic Mpc complexes. Samples were tested by applying BEST-TROSY experiments. After that, MTSL was reduced by VC and samples were tested using BEST-TROSY experiments again to obtain diamagnetic Mpc complexes.

The intensities of roughly nine peaks of the ^15^N-Mpc1/MTSL–Mpc1 complex were reduced (green ellipse markers) (Figure 4A), and six of these peaks underwent complete broadening, showing that the MTSL-labeled C87 interacted closely with the surrounding amino acids [20]. However, complete broadening cannot happen when distance exceeds 12 Å [28], the micelle’s monomer LDAO’s length is around 20.7 Å [29] and the other component’s DPC is about 20.3 Å. Thus, C87 was in the same micelle as a paramagnetic-relaxation-enhanced amino acid of the other Mpc1 monomer. Therefore, Mpc1 monomers interact.

Meanwhile, in the BEST-TROSY spectrum of ^15^N-Mpc1/MTSL–Mpc2 (Figure 4B), about fifteen peaks declined in intensity, and about nine of them underwent complete broadening, indicating that the MTSL-labeled Mpc2 C86 and C111 were in very close proximity to the surrounding Mpc1. In the ^15^N-Mpc2/MTSL–Mpc1 complex (Figure 4D), the MTSL-labeled Mpc1 C87 affected about six peaks, and these six peaks underwent complete broadening. This indicates that Mpc1 C87 and some amino acids of Mpc2 are in proximity, are in the same micelle, and interact with each other. A similar phenomenon was detected in ^15^N-Mpc2/MTSL–Mpc2 (Figure 4C), where roughly five peaks underwent complete peak broadening; it may be deduced that the nearby relaxation-enhanced amino acids of the other Mpc2 monomer interact with the Mpc2 C86 and C111. It may be possible to infer interactions between the two monomers of the Mpc1 homo-dimer, Mpc2 homo-dimer and Mpc1–Mpc2 hetero-dimer.

### 3.4. Thermostability of the Mpc Complexes

**Figure 5:**
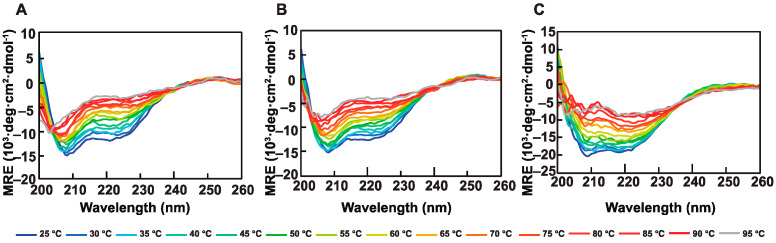
Thermostability analysis of the Mpc complexes using far-ultraviolet CD. (**A**) CD of Mpc1^linker^ complex at various temperatures (from 25 to 95 °C); (**B**) CD of Mpc1^linker^–Mpc2 complex at various temperatures (from 25 to 95 °C); (**C**) CD of Mpc2 complex at various temperatures (from 25 to 95 °C). The temperature of each curve is presented below the spectra. The high-tension voltage (HT voltage) exceeded 800 V, causing a distortion of data from 190 to 200 nm as the temperature increased. As a result, CD curves for wavelengths ranging from 190 to 200 nm were not included in the analysis.

The thermal stability of Mpc homo- and hetero-dimers were examined by CD. With temperature being ramped in intervals of 5 °C from 25 to 95 °C, wavelength scans were carried out from 200 to 260 nm. In the variation of CD curves during the process of temperature change, the three complexes displayed distinctive patterns: for Mpc1^linker^ homo-dimer, the CD curves were relatively sparse in its low-temperature zone (from 25 to 65 °C) and were dense in the high-temperature zone (from 70 to 95 °C) (Figure 5A); for the Mpc2 homo-dimer, the CD curves were sparse in the intermediate-temperature zone between 35 and 85 °C while they were dense at both ends (Figure 5C); for the Mpc1^linker^–Mpc2 hetero-dimer, the CD curves showed greater uniformity than those of the homo-dimers (Figure 5B), and this variation could possibly be a result of various structural makeups.

To learn more about how stable the three complexes were, the area between the two CD curves at 25 and 95 °C (Figure 5A–C) was integrated. This showed how big the overall CD variation in the three complexes was. As shown in Table 1, the Mpc2 complex had the biggest overall CD variation in this temperature range, no matter which spline interpolation method was used (linear, cubic and quadratic) (Figure S3B–D). To determine whether or not the overall changes (Figure S3A) in CD values of the three complexes were statistically different, the Friedman nonparametric hypothesis test and Kendall’s coefficient of concordance were applied. With a *p*-value of 0.019, it was apparent that there were statistically significant differences among the three Mpc complexes. Moreover, there were statistically significant differences between the Mpc2 homo-dimer and another two complexes (*p* = 0.044 < 0.05). The Mpc1^linker^ homo-dimer and the Mpc1^linker^–Mpc2 hetero-dimer, however, did not differe statistically significantly (*p* = 1 > 0.05), which also showed the high similarity of the Mpc1^linker^ homo-dimer to the Mpc1^linker^–Mpc2 hetero-dimer in thermostability, suggesting that Mpc1 may play a more significant role in the overall stability of protein.

In conclusion, the thermal stability of the Mpc2 homo-dimer was inferior to that of the Mpc1^linker^ homo-dimer, and that of the latter was slightly inferior to that of the Mpc1^linker^–Mpc2 hetero-dimer.

### 3.5. Transport Functional Verification of Mpc Complexes

**Figure 6:**
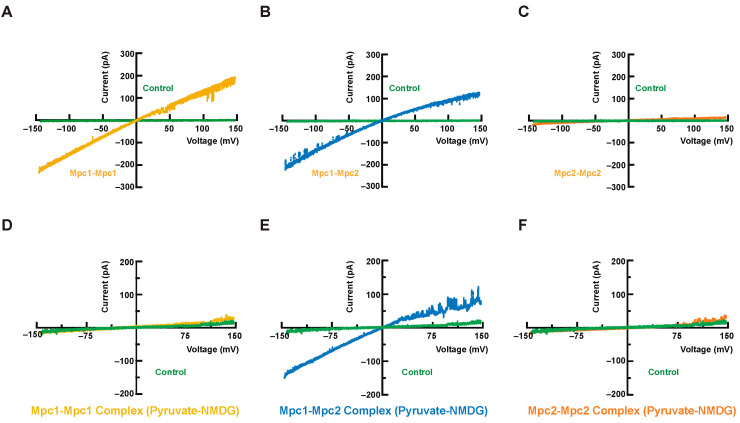
Transportation of small ions (potassium ions) and pyruvate. (**A**–**C**) current–voltage relations of the Mpc1 homo-dimer (**A**) (in rattan yellow), the Mpc1–Mpc2 hetero-dimer (**B**) (in blue), the Mpc2 homo-dimer (**C**) (in orange), and the empty liposome (in green). The solution here is 10 mM Mops (pH 7.2) and 250 mM KCl; (**D**–**F**) the channel current–voltage relations of the Mpc1 homo-dimer (**D**) (in rattan yellow), the Mpc1–Mpc2 hetero-dimer (**E**) (in blue), the Mpc2 homo-dimer (**F**) (in orange), and the empty liposome (in green). The solution here contained pyruvate-N-Methyl-D-glucamine (NMDG) and Mops only.

We conducted single-channel patch-clamp assays to verify whether or not Mpc complexes can transport ions. Compared to the empty liposome control (green curves), the Mpc1 homo-dimer (Figure 6A) and the Mpc1–Mpc2 hetero-dimer (Figure 6B) exhibited higher current signals. However, Mpc2 homo-dimer exhibited no detectable current signal (Figure 6C). This phenomenon suggested that the Mpc1 homo-dimer and the Mpc1–Mpc2 hetero-dimer could transport K^+^ ions in the pipette buffer, indicating the formation of a channel-like structure.

A second set of pyruvate-NMDG single-channel patch-clamp assays were designed to find out if the Mpc complexes could transport pyruvates (Figure 6D–F). The Mpc1–Mpc2 hetero-dimer could continue to sustain a reasonably high current signal under the same voltage mode, but neither the Mpc1 homo-dimer nor the Mpc2 homo-dimer could. NMDG has been widely used as an impermeant cation in patch-clamp experiments as a blank reference [24]; Mpc1–Mpc2 complexes herein are capable of pyruvate transport. Compared to the K^+^ ion environment, the current signal intensity of the Mpc1–Mpc2 hetero-dimer was noticeably weaker in the NMDG environment. These dissimilarities may be explained by the fact that ions with a larger ionic radius and lower charge density produce weaker current signals with the same voltage.

## 4. Discussion

Mpc complexes are the key inner mitochondrial membrane proteins, which are related to the transport of mitochondrial pyruvates. There has been some controversy over the functional reconstitution and oligomeric state of Mpc proteins. Furthermore, little has been known about interactions among subunits, together with the structure of Mpc complexes.

To obtain conclusive evidence for the aforementioned issues, the first challenge is to successfully express, purify, and reconstitute individual Mpc proteins in a suitable membrane environment. Great efforts have been made. Bender et al. expressed yeast MPCs fused C-terminally to a 3×HA-tag from yeast endogenous promoters, but the states of monomers, dimers and higher oligomers are determined by chemical cross-linking methods [8]. Human Mpc1 and Mpc2 were co-expressed in a yeast system or individually overexpressed in sf9 cells with relatively complicated procedures [15]. Because MPCs are small proteins, it is possible to simply express them in the prokaryotic expression system. Li et al. tried to produce recombinant yeast Mpc proteins by introducing fusing protein, maltose-binding protein, and cyanogen cleavage, through which stable Mpc monomers were successfully obtained [27]. Herein, we utilized the relatively simple prokaryotic expression system without complicated procedures. Moreover, homo- and hetero-dimers were successfully assembled into the mixed detergent micelles.

Interactions among subunits of Mpc complexes were rarely reported. Here, in this study, multiple NMR methods were applied to investigate such interactions. ^1^H-^15^N BEST-TROSY spectra were used to record changes in chemical shifts and in intensities between samples of pure ^15^N-Mpc1 and ^15^N-Mpc1/Mpc2 complexes. Observable alterations were documented, indicating that the states of Mpc1 were dissimilar in the presence or absence of Mpc2. A similar phenomenon was detected for Mpc2 with or without Mpc1. PRE NMR is a popular technique used for studying complexes and membrane proteins. It involves introducing a paramagnetic species to the protein of interest, inducing local relaxation rate changes in the surrounding nuclei that can be detected by NMR [30]. PRE NMR provides valuable long-range structural and interactional information in large and complex protein systems where traditional NMR methods may be limited. This is particularly important for studying membrane protein complexes, which are challenging to study using solution NMR due to their hydrophobic nature and complex membrane environment. In this study, we used PRE NMR with the MTSL probe in Mpc1 to detect PRE effects on Mpc2 and vice versa. Apparent PRE effects were discovered with a sample containing unlabeled MTSL–Mpc2 and ^15^N–Mpc1 at a ratio of 1:1, as well as unlabeled MTSL–Mpc1 and ^15^N–Mpc2. Collectively, the NMR results validated the interactions between Mpc monomers.

Confirming the oligomeric state of Mpc complexes is essential for pyruvate transport mechanism and structure determination, although there is long-standing acceptance that Mpc1 and Mpc2 can form a multi-dimer with a molecular weight of about 150 kDa on blue native gels [1]. However, some reports indicated that the functional states of Mpc complexes are hetero-dimers and also clarified that the molecular weight of these complexes may be overestimated when accounting for the effects of membrane lipids or detergents on blue native gels [16]. In addition to hetero-complexes, homo-complexes were also proven to have bioactivities in different cells [12,13,14]. In this report, several biochemical experiments were also performed, and we confirmed that Mpc complexes can form hetero-dimers as well as homo-dimers.

Interestingly, both the Mpc1 homo-dimer and Mpc1–Mpc2 hetero-dimer in a giant liposome can transport K^+^ ions by single-channel patch-clamp assays while the Mpc2 homo-dimer lacks this ability. These findings support the importance of the Mpc1 monomer for pyruvate transport in yeast cells, as previously reported [8,16]. However, only the Mpc1–Mpc2 heterodimer can transport pyruvate, which may be due to the influence of Mpc2 on the conformation of Mpc1.The functional assay results collected by single-channel patch-clamp assays reveal that (1) the Mpc1–Mpc2 hetero-dimer is the core component of Mpc complexes and (2) Mpc complexes are more likely to form a channel rather than act as a facilitative carrier.

In this study, we demonstrated that *E. coli* recombinant expression is viable for obtaining individual Mpc proteins. Interactions among subunits of Mpc complexes were investigated by PRE NMR methods. We successfully obtained not only Mpc1 homo-dimers but also Mpc1–Mpc2 hetero-dimers, and confirmed that both can transport K^+^ ions by single-channel patch-clamp assays. However, the Mpc1–Mpc2 hetero-dimer was the sole complex that could transport pyruvate. Our study has paved an important path towards the determination of the structure of Mpc complexes.

## Figures and Tables

**Table 1:** *d-value analysis of thermostabilities of Mpc complexes.

Protein Complex	Original d-Value	Spline Interpolation Method		
		Linear	Cubic	Quadratic
	Curve Change Area (deg m^3^·(3 × 10^9^ mol)^−1^)			
Mpc1^linker^ homo-dimer	222.828	220.054	222.062	220.342
Mpc1^linker^–Mpc2 hetero-dimer	213.457	208.063	208.229	208.219
Mpc2 homo-dimer	267.816	259.925	260.230	260.297

*d-value denotes the residuals in data points between the 95 and 25 °C CD curves.

## Data Availability

Not applicable.

## Supplementary Materials

The following supporting information can be downloaded at https://www.mdpi.com/article/10.3390/biom13050719/s1. Figure S1: BEST-TROSY spectra of Mpc complexes; Figure S2: proportion of secondary structure present in Mpc complexes; Figure S3: spline interpolation method for the residual curves between CD curves of 95 and 25 °C.
