# Supplementary material for: NMR and Patch-Clamp Characterization of Yeast Mitochondrial Pyruvate Carrier Complexes
Source: Biomolecules. 2023 Apr 22;13(5):719. doi: 10.3390/biom13050719 (PMC10216714; doi:10.3390/biom13050719)
Supplement: Supplementary file 1 [file biomolecules-13-00719-s001.zip › biomolecules-2275523-supplementary.pdf]

biomolecules

MDPI

Article

NMR and Patch-Clamp Characterization of Yeast Mitochondrial Pyruvate Carrier Complexes

Zhen Wang [1,2], Wen Ding [3], Maosen Ruan [1], Yong Liu [1,2], Jing Yang [1], Huiqin Zhang [1,4], Bing Shen [3], Junfeng Wang [1,2,4,*] and Yunyan Li [1,*]

1 High Magnetic Field Laboratory, CAS Key Laboratory of High Magnetic Field and Ion Beam Physical Biology, Hefei Institutes of Physical Science, Chinese Academy of Sciences, Hefei 230031, China
2 Hefei Institutes of Physical Science (Branch of Graduate School), University of Science and Technology of China, Hefei 230026, China
3 School of Basic Medical Sciences, Anhui Medical University, Hefei 230032, China
4 Institutes of Physical Science and Information Technology, Anhui University, Hefei 230601, China
* Correspondence: junfeng@hmfl.ac.cn (J.W.); yyli@hmfl.ac.cn (Y.L.)

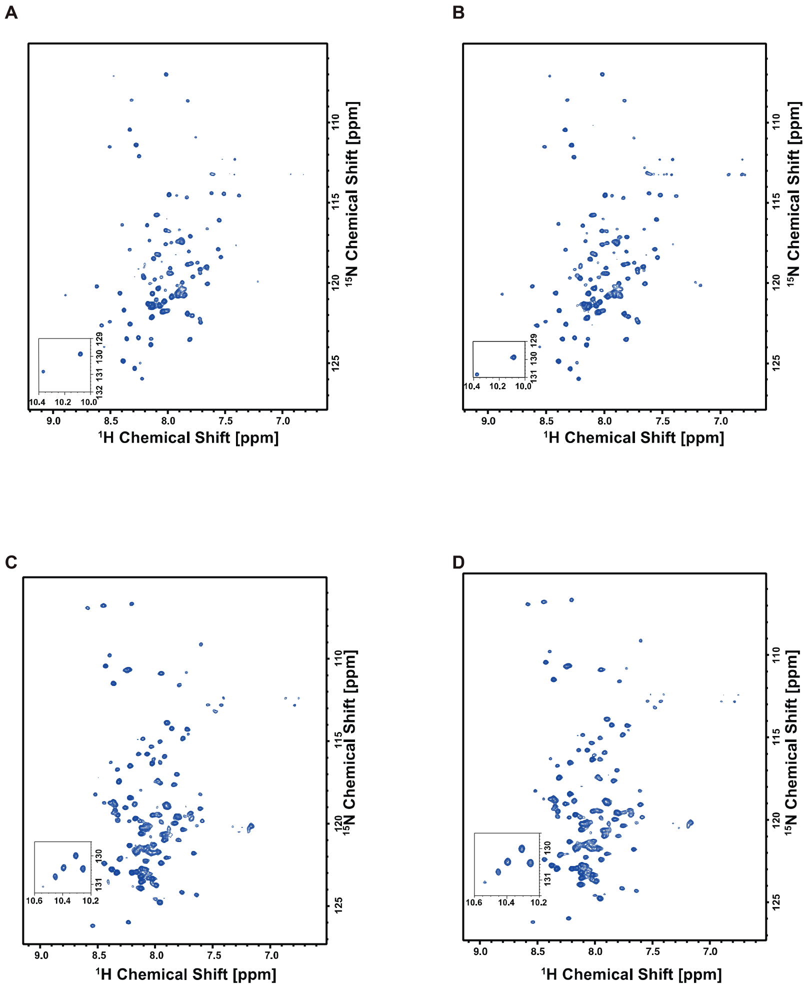

Figure S1. BEST-TROSY spectra of Mpc complexes. The peaks of aromatic amino acids were placed in separate boxes located in the lower left corner of each spectrum: (**A**) BEST-TROSY spectrum of ^{15}N Mpc1-Mpc1 Complex; (**B**) BEST-TROSY spectrum of ^{15}N Mpc1-Mpc2 Complex; (**C**) BEST-TROSY spectrum of ^{15}N Mpc2-Mpc1 Complex; (**D**) BEST-TROSY spectrum of ^{15}N Mpc2-Mpc2 Complex.

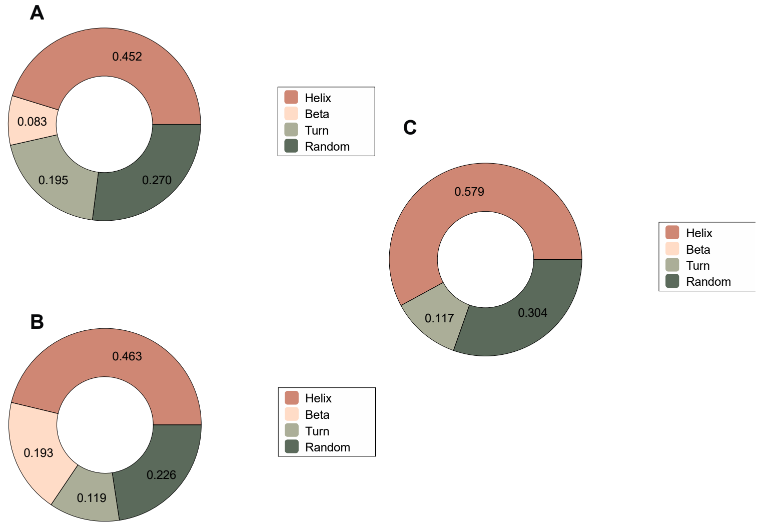

Figure S2. The proportion of secondary structure present in Mpc complexes. (**A**) Secondary structure proportion of Mpc1^{linker}-Mpc1^{linker}; (**B**) Secondary structure proportion of Mpc1^{linker}-Mpc2; (**C**) Secondary structure proportion of Mpc2-Mpc2.

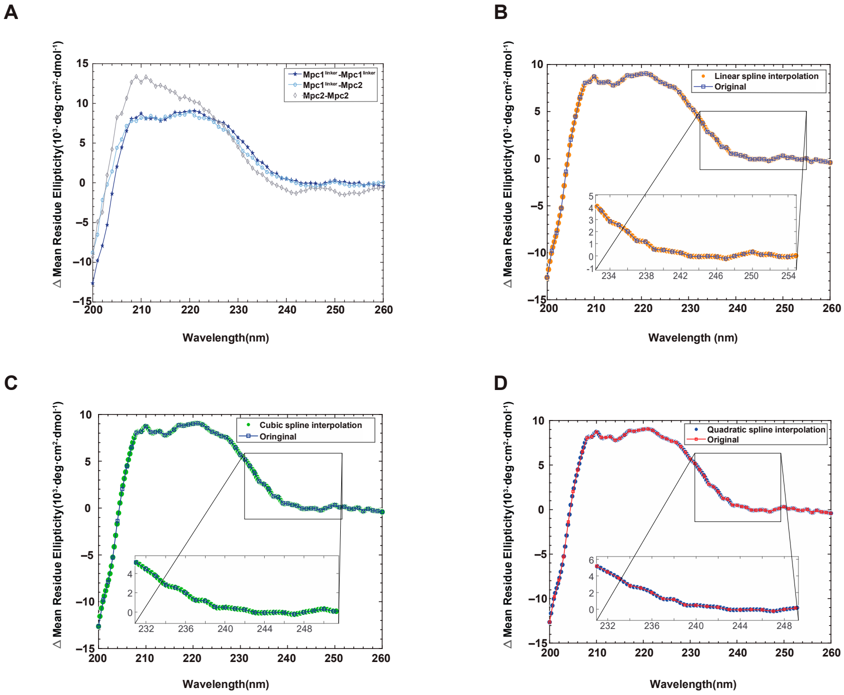

Figure S3. Spline interpolation method of the residual curves between CD curves of 95 ℃ and 25 ℃. The introduction of spline interpolation is necessary for the calculation of the area between two curves when there are fewer data points than required. The last three (**B**–**D**) were cases of Mpc1^{linker}-Mpc1^{linker} to illustrate this method. The portion enclosed by the rectangular box in every single subfigure is the section between the two intersections of 25 ℃ CD curve and 95 ℃ CD curve : (**A**) Residual curves of three Mpc complexes; (**B**) Linear spline interpolation of the residual curve; (**C**) Cubic spline interpolation of the residue curve; (**D**) Quadratic spline interpolation of the residual curve.
